# Influence of the dissipation on the N-level atom interacting with a two two-level atoms in presence of qubit–qubit interaction

**DOI:** 10.1038/s41598-021-85944-6

**Published:** 2021-04-01

**Authors:** S. Abdel-Khalek, Hashim M. Alshehri, E. M. Khalil, A.-S. F. Obada

**Affiliations:** 1 Department of Mathematics, College of Science, Taif University, P.O. Box 11099, Taif, 21944 Saudi Arabia; 2 Mathematics Department, Faculty of Science, Sohag University, Sohag, Egypt; 3 Mathematics Department, Faculty of Science, King Abdulaziz University, Jeddah, 21521 Saudi Arabia; 4 Mathematics Department, Faculty of Science, Al-Azhar University, Nasr City, Cairo, 11884 Egypt

**Keywords:** Applied mathematics, Quantum information, Qubits

## Abstract

The interacting of two qubits and an N-level atom based on su(2) Lie algebra in the presence of both qubit–qubit interaction and dissipation term is considered. The effects of the qubit–qubit interaction and the dissipation term on the dynamics of the proposed system are discussed in detail for certain values of the number of levels. The dynamical expressions of the observable operators are obtained using the Heisenberg equation of motion. The population inversion and linear entropy, as well as the concurrence formula as a measure of entanglement between the two qubits are calculated and discussed. The roles of the number of levels, the qubit–qubit coupling parameter and the dissipation rate on these quantities are also discussed. We explore the sudden birth and sudden death of the entanglement phenomena with and without the dissipation term.

## Introduction

The open quantum systems have abundant scientific applications such as^1–3^. A system cannot be found alone. Rather, it is with other degrees of freedom that are indicated as the environment. Due to the large number of applications, the study of the open quantum system under various types of damping effect has attained great attention concerning the impacts of the dynamical behavior of different quantum phenomena^3^ . To explore the impact of the environment on quantum phenomena, especially quantum correlation the operator-sum representation is adopted. Although we innately suppose that the environment can spoil the quantum signature and the correlation of the system, studying the dynamics of this process in detail to process realistic information is significant. Memory impacts the dynamics of open quantum systems in the light of the quantum Zeno effect play a role^4^. It is demonstrated that the exploitation of the analogy between quantum and dissipation measurements and the interaction between the system and the matter promote quantum Zeno dynamics. Furthermore, the change of the measurement parameters may tune the dissipation strength that the system experiences. The open quantum system with a nonequilibrated environment that consists of numerous non-Markovian reservoirs suggests an emergent thermal behavior pseudo under the thermalization influence was explored^5^. The obtained results clarified that having a dispersive environment or a saturable pumping breaks down the pseudo thermalization impact. Additionally, Iotti and Rossi discussed the effect of energy dissipation and decoherence in solid-state quantum devices in the framework of Markovian against non-Markovian treatment type schemes^6^. The effects of dissipation and thermal noise on hybrid open quantum systems via the ultrastrong-coupling system were examined by Settineri et al.^7^.

A primary goal of quantum technologies is to control the quantum correlations between subsystems^8–10^. This quantum correlation was studied for each qubit in a system with two atoms and the environment concerning the interatomic distance^11^. Recently, various devices, e.g., beam splitters^12–14^, nanoresonators^15^, interactions in superconducting circuit-QED systems^16,17^ and optomechanical interactions^18^ have been developed to obtain quantum entanglement. All entanglement measures should be stable according to local operations in classical communication. The entanglement of formation is an entanglement measure for bipartite quantum states while VNE is an optimal measure of entanglement on a pure state^19^. Consequently, researchers utilized various statistical aspects to explore the different correlations of a quantum system. In this regard, the relationship between the quantum Fisher information and a nonlocal correlation indicates the entanglement of the quantum system^20^. Important physical phenomena, e.g., entanglement sudden birth (ESB) and entanglement sudden death (ESD) between bipartite systems were explored^21,22^. Moreover, entanglement between two coupled three-level atoms for different types of time-dependent coupling was investigated. The phenomena of ESD and ESB were found to depend on the kind of time-dependent coupling between the two atoms^23^.

The main direction in the technology of quantum information is the generalization of the different kinds of interaction between quantum systems. Thus, the explored models provided new physical settings and became more realizable when having the decoherence effect. Thus the analyses of phenomena exhibiting finite-time decay of entanglement have recently attracted considerable attention. Also, the energy dissipation leads to decreasing or losing the entanglement between quantum systems. It is shown that considerable phenomena such as entanglement sudden death^1,24^, entanglement revival^25^, etc. The dissipation of an atomic-field system has been introduced by^2,26,27^. Also, the dissipation in systems of linear and nonlinear quantum scissors has been investigated^28^. Moreover, the influence of phase of the coupling on entanglement decay in the nonlinear coupler system has been studied^29^. It is shown that the occurrence of sudden death and sudden birth phenomena can be controlled by the coupling phase parameter. Bartkowiak et al analyzed different finite-time decays and analogous periodic vanishing of nonclassical correlations as described by violations of classical inequalities and the corresponding quantumness witnesses^30^. It is shown that these sudden vanishings are universal phenomena and can be observed in the case of single-mode, two-mode and multimode fields. Hence, the present paper aims to explore and understand the influences of the dissipation on the N-level atom that interacts with two two-level atoms in the presence of atom-atom interaction. In addition, we explore the link between the linear entropy, atomic inversion and qubit–qubit entanglement. The contents of the article are arranged as follows: We present the general solution of the proposed system under dissipation effect in section “Description of the model and solution”. The numerical results of the population inversion in section “Population inversion”. Section “Linear entropy” will discuss the dynamics of the linear entropy in addition to the qubit–qubit entanglement measured by the concurrence. Finally, we conclude our results in section “Conclusion”.

## Description of the model and solution

An application of the su(2) algebraic system has been presented in^31,32^. They examined the star network of spins, where all rotations interact exclusively and continuously with a central rotation through Heisenberg XX couplings of equal strength. They considered that the spin star as central qubit interacts with all other qubits, and the non-central qubits do not interact directly with each other. Therefore, the quantum system that represents this phenomenon is given as follows,

1 $$\begin{aligned} \frac{\hat{H}}{\hbar }=\frac{\omega }{2}\sum _{j=1}^{N}\pi _{z}^{j}+\sum _{k=1}^{2}\left[ \Omega _{1}^{k}{\hat{S}}_{11}^{k}+\Omega _{2}^{k} {\hat{S}}_{22}^{k}+\lambda ({\hat{S}}_{12}^{k}\sum _{j=1}^{N}\pi _{-}^{j}-{\hat{S}} _{21}^{k}\sum _{j=1}^{N}\pi _{+}^{j})\right] +{\mathrm {iR}}\left( {\hat{S}} _{12}^{(1)}\ {\hat{S}}_{21}^{(2)}-{\hat{S}}_{21}^{(1)}{\hat{S}}_{12}^{(2)}\right), \end{aligned}$$

The parameters $\omega$ and $\Omega _{1}^{k},\Omega _{2}^{k}$ are respectively the frequencies of the central qubit and the surrounding ones, while $\lambda$ is the coupling parameter, $R$represents the coupling between qubits themselves. The ${\hat{S}} _{kl}^{k}$ and $\pi _{z}^{j}$ denote the generators of operators of su(2) which are obey the commutation relation:

$$[{\hat{S}}_{kl}^{j},{\hat{S}}_{mn}^{i}]=\delta _{lm}{\hat{S}}_{kn}^{j}.$$

We add the dissipation term and use the collective operator $\hat{J} _{k}=\sum _{j=1}^{N}\pi _{k}^{j},\ (k=z,+,-).$ Therefore, equation (1) becomes,

2 $$\begin{aligned} \frac{\hat{H}}{\hbar }=(\omega -i\gamma )\hat{J}_{z}+\sum _{k=1}^{2}\left[ \Omega _{1}^{k}{\hat{S}}_{11}^{k}+\Omega _{2}^{k}{\hat{S}}_{22}^{k}+\lambda ( {\hat{S}}_{12}^{k}\hat{J}_{-}-{\hat{S}}_{21}^{k}\hat{J}_{+})\right] +{\mathrm {iR}}\left( {\hat{S}}_{12}^{(1)}\ {\hat{S}}_{21}^{(2)}-{\hat{S}}_{21}^{(1)}{\hat{S}} _{12}^{(2)}\right), \end{aligned}$$

where $\gamma$ denotes the decay rate,  $\hat{J}_{-}$ $,\hat{J}_{+}$ denote the lowering and raising operators of the su(2) symmetry and satisfy the following commutation relation:

3 $$[\hat{J}_{-},\hat{J}_{+}]=-2\hat{J}_{z},\quad [\hat{J}_{z}, \hat{J}_{\pm }]=\pm \hat{J}_{\pm }.$$

For describes and explains the physical phenomena for the model (2 ), we write the differential equations by applying the Heisenberg equations of motion (HEM). Therefore, the mathematical expressions of the HEM take the form

4 $$\begin{aligned} \frac{d\hat{J}_{z}}{dt}= & i\lambda ({\hat{S}}_{12}^{1}\hat{J}_{-}-{\hat{S}} _{21}^{1}\hat{J}_{+})+i\lambda ({\hat{S}}_{12}^{2}\hat{J}_{-}-{\hat{S}}_{21}^{2} \hat{J}_{+}), \nonumber \\ \frac{d{\hat{S}}_{11}^{k}}{dt}= & -i\lambda ({\hat{S}}_{12}^{k}\hat{J}_{-}-\hat{S }_{21}^{k}\hat{J}_{+})+R\left( {\hat{S}}_{12}^{(1)}\ {\hat{S}}_{21}^{(2)}+{\hat{S}} _{21}^{(1)}{\hat{S}}_{12}^{(2)}\right) \nonumber \\ \frac{d{\hat{S}}_{22}^{k}}{dt}= & i\lambda ({\hat{S}}_{12}^{k}\hat{J}_{-}-{\hat{S}} _{21}^{k}\hat{J}_{+})-R\left( {\hat{S}}_{12}^{(1)}\ {\hat{S}}_{21}^{(2)}+{\hat{S}} _{21}^{(1)}{\hat{S}}_{12}^{(2)}\right) ,\ k=1,2 \end{aligned}$$

from which we can show that,

5 $$\begin{aligned} \hat{C}=\hat{J}_{z}+\sum _{k=1}^{2}\left( \frac{{\hat{S}}_{11}^{k}-{\hat{S}} _{22}^{k}}{2}\right) , \end{aligned}$$

where $\hat{C}$ is a constant of motion. Therefore, the Hamiltonian system ( 2) becomes,

6 $$\begin{aligned} \frac{\hat{H}}{\hbar }=(\omega -i\gamma )\hat{C}+\hat{D} \end{aligned}$$

where the operator $\hat{D}$ takes the form

7 $$\begin{aligned} \hat{D}=\sum _{k=1}^{2}\left( \delta _{z}^{k}\left[ \frac{{\hat{S}}_{11}^{k}- {\hat{S}}_{22}^{k}}{2}\right] +\lambda ({\hat{S}}_{+}^{k}\hat{J}_{-}+{\hat{S}} _{-}^{k}\hat{J}_{+})\right) +{\mathrm {iR}}\left( {\hat{S}}_{12}^{(1)}\ {\hat{S}} _{21}^{(2)}-{\hat{S}}_{21}^{(1)}{\hat{S}}_{12}^{(2)}\right) \end{aligned}$$

the quantities $\mathrm {\delta }_{z}^{k}$ denote the detuning function which can be expressed as

8 $$\begin{aligned} \mathrm {\delta }_{z}^{k}=(\Omega _{1}^{k}-\Omega _{2}^{k}-\omega )+i\gamma ,\quad \text {where}\quad k=1,2. \end{aligned}$$

The general solution for the two-qubit is obtained by calculating the time evolution operator as follows,

9 $$\begin{aligned} \hat{U}(t)=\exp (-i\hat{H}t)=\exp (-i(\omega -i\gamma )\hat{C}t)\otimes \exp (-i\hat{D}t) \end{aligned}$$

Under the exact resonance case condition $(\mathrm {\delta }_{z}^{1}\mathrm { +\delta }_{z}^{2}\mathrm {=0})$ and for space of the two qubits as $|ee\rangle$, $|eg\rangle$, $|ge\rangle$ and $|gg\rangle$ the analytical form of the time evolution operator $\hat{U}(t)$ is given by

10 $$\begin{aligned} {} &\exp (-i\hat{D}t)=\left[ \begin{array}{llll} 1+\hat{J}_{+}\hat{\Gamma }\hat{J}_{-} & \frac{\delta ^{*}}{4\lambda }\hat{ J}_{+}\hat{\Gamma }-i\hat{J}_{+}\hat{\Lambda } & \frac{-\delta ^{*}}{ 4\lambda }\hat{J}_{+}\hat{\Gamma }-i\hat{J}_{+}\hat{\Lambda } & \hat{\Gamma } ^{\dagger }\hat{J}_{-}\hat{\Gamma }^{\dagger } \\ \frac{\delta }{4\lambda }\hat{\Gamma }\hat{J}_{-}-i\hat{\Lambda }\hat{J}_{-} & \frac{\hat{\eta }_{1}^{2}}{2\lambda ^{2}}\hat{\Gamma }-\frac{i\delta }{ 2\lambda }\hat{\Lambda }+1 & \frac{(\hat{J}_{+}\hat{J}_{-}+\hat{J}_{-}\hat{J} _{+})}{2}\hat{\Gamma } & \frac{\delta }{4\lambda }\hat{\Gamma }\hat{J}_{+}-i \hat{\Lambda }\hat{J}_{+} \\ \frac{-\delta }{4\lambda }\hat{\Gamma }\hat{J}_{-}-i\hat{\Lambda }\hat{J}_{-} & \frac{(\hat{J}_{+}\hat{J}_{-}+\hat{J}_{-}\hat{J}_{+})}{2}\hat{\Gamma } & \frac{\hat{\eta }_{1}^{2}}{2\lambda ^{2}}\hat{\Gamma }+\frac{i\delta ^{*}}{ 2\lambda }\hat{\Lambda }+1 & \frac{-\delta ^{*}}{4\lambda }\hat{\Gamma } \hat{J}_{+}-i\hat{\Lambda }\hat{J}_{+} \\ \hat{J}_{-}\hat{\Gamma }\hat{J}_{-} & \frac{\delta ^{*}}{4\lambda }\hat{J} _{-}\hat{\Gamma }-i\hat{J}_{-}\hat{\Lambda } & \frac{-\delta }{4\lambda }\hat{J }_{-}\hat{\Gamma }-i\hat{J}_{-}\hat{\Lambda } & 1+\hat{J}_{-}\hat{\Gamma }\hat{J }_{+} \end{array} \right] \nonumber \\&\exp (-i(\omega -i\gamma )\hat{C}t)=\exp -\hat{J}_{z}(\gamma +i\omega )t \left[ \begin{array}{llll} \exp -(\gamma +i\omega )t & 0 & 0 & 0 \\ 0 & 1 & 0 & 0 \\ 0 & 0 & 1 & 0 \\ 0 & 0 & 0 & \exp (\gamma +i\omega )t \end{array} \right] \end{aligned}$$

where

11 $$\begin{aligned} \hat{\Gamma }= & \frac{2\lambda ^{2}(\cos \hat{\eta }_{2}t-1)}{\hat{\eta } _{2}^{2}},\ \hat{\Lambda }=\frac{\lambda \sin \hat{\eta }_{2}t}{\hat{\eta }_{2}} \nonumber \\ \hat{\eta }_{2}^{2}= & \frac{|\delta |^{2}}{4}+2\lambda ^{2}(\hat{J}_{+}\hat{J }_{-}+\hat{J}_{-}\hat{J}_{+}),\ \hat{\eta }_{1}^{2}=\frac{|\delta |^{2}}{4} +\lambda ^{2}(\hat{J}_{+}\hat{J}_{-}+\hat{J}_{-}\hat{J}_{+}) \nonumber \\ \delta= & \delta _{z}^{1}+iR \end{aligned}$$

Now we assume that the initial conditions for the present system (2 ) is set as follows, the two qubits in their the excited states, while the N-level atom starts from the atomic coherent state, which is given as follows,

12 $$\begin{aligned} |\psi (0)\rangle =|ee\rangle \otimes |\beta \rangle , \end{aligned}$$

where, $|\beta \rangle$ can be expressed as the following,

13 $$\begin{aligned} |\beta \rangle =\sum _{n=-j}^{j}Q_{n}^{j}(\Theta ,\Phi )|n,j\rangle , \end{aligned}$$

the coupling coefficients $Q_{n}^{j}(\Theta ,\Phi )$ is obey the following relation,

14 $$\begin{aligned} Q_{n}^{j}=\sqrt{C_{j+n}^{2j}}e^{i(j-n)\Phi }\left( \cos \frac{\Theta }{2} \right) ^{j+n}\left( \sin \frac{\Theta }{2}\right) ^{j-n}, \end{aligned}$$

where $C_{j+n}^{2j}$ is the binomial coefficient, $\Theta \in [0,\pi ]$ and $\Phi \in [0,2\pi ]$. The exact solution $|\Psi (t)\rangle$ for $t>0$ is

15 $$\begin{aligned} |\Psi (t)\rangle= & \hat{U}(t)|\psi (0)\rangle \nonumber \\= & \sum _{n=-j}^{j}[Y_{1}(n,t)|ee\rangle |n,j\rangle +Y_{2}(n,t)|eg\rangle |n+1,j\rangle +Y_{3}(n,t)|ge\rangle |n+1,j\rangle \nonumber \\&+Y_{4}(n,t)|gg\rangle |n+2,j\rangle ]. \end{aligned}$$

The functions *Y* $_{1},Y_{2},Y_{3}$ and *Y* $_{4}$ are given by

16 $$\begin{aligned} {Y_{1}(n,t)}= & Q_{n}^{j}\exp \left\{ -(n+1)(\gamma +i\omega )t\right\} \left[ \frac{(\mu _{1}(n))^{2}-2\nu _{1}^{2}(n)(1-\cos \mu _{1}(n)t)}{(\mu _{1}(n))^{2}}\right] , \nonumber \\ Y_{2}(n,t)= & -Q_{n+1}^{j}\exp \left\{ -n(\gamma +i\omega )t\right\} \left[ \frac{\delta \ \nu _{1}(n)(1-\cos \mu _{1}(n)t)}{(\mu _{1}(n))^{2}}+i\frac{ \nu _{1}(n)\sin \mu _{1}(n)t}{\mu _{1}(n)}\right] , \nonumber \\ Y_{3}(n,t)= & -Y_{2}^{*}(n,t), \nonumber \\ {\ Y_{4}(n,t)}= & -Q_{n+2}^{j}\exp \left\{ (1-n)(\gamma +i\omega )t\right\} \left[ \frac{2\nu _{1}(n)\nu _{2}(n)(1-\cos \mu _{1}(n)t)}{(\mu _{1}(n))^{2}}\right] , \nonumber \\ v_{1}(n)= & \lambda \sqrt{(j-n)(n+j+1)}\text {,}v_{2}=\lambda \sqrt{ (j-n+1)(n+j)}, \nonumber \\ \mu _{1}(n)= & \sqrt{\left| \delta \right| ^{2}+2[\nu _{1}(n)]^{2}+2[\nu _{2}(n)]^{2}}. \end{aligned}$$

After obtained on the wave function (15) can be used to describe the evolution of some quantum quantifiers as: population inversion, linear entropy and concurrence.

## Population inversion

**Figure 1:**
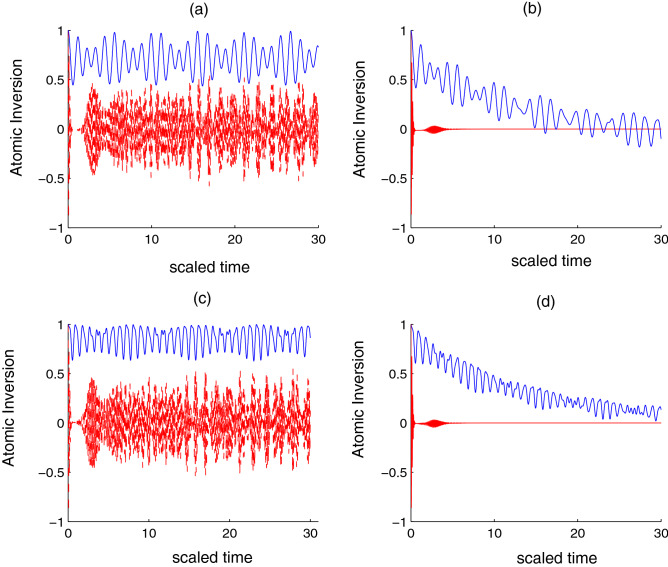
Time evolution of the *atomic inversion* *W*(*t*) for $\Theta = \pi /2$ and $\Phi =\pi /4$. (**a**) is for ($j=2$ blue line, $j=40$ red line and the absence of atom-atom coupling $R=0$). (**c**) is for (red line $j=2$, red line $j=40$, and the presence of atom-atom coupling $R=5\lambda$. (**b**,**d**) are the same as (**a**,**c**) under the field dissipation effect rate $\gamma =0.01\lambda.$

The population inversion is one of the important quantities in quantum information. Based on its results, we can determine the periods of collapse and revival, which are useful in determining the periods of maximally entangled state and purity periods (separable state)^33,34^. The population inversion function is identified as the difference between the probability of finding the qubit in the excited and the ground states. When the qubit begins in the excited states, the population inversion takes the form of

17 $$\begin{aligned} W(t)= & \rho _{11}(t)-\rho _{44}(t) \nonumber \\ \rho _{11}(t)= & \sum _{n=-j}^{j}\left| {Y_{1}(n,t)}\right| ^{2},\ \rho _{44}(t)=\sum _{n=-j}^{j}\left| {Y_{4}(n,t)}\right| ^{2} \end{aligned}$$

First, we consider the two qubits in their excited states initially and exclude both of the qubit–qubit interaction ($R=0$) and the dissipation term ($\gamma =0$). In the case of $j=2$, the population inversion ranges randomly from 0.5 to 1. That is, the population inversion almost occurs in the excited state. Additionally, the phenomena of collapse and revival does not appear. After increasing the number of levels to 40, the *W*(*t*) function fluctuates between $-0.5$ to 0.5. Moreover, the previous random form becomes somewhat regular and periodic. A single period of collapses and revivals appears clearly, as shown in Fig. 1a. When the dissipation of the interaction cavity is considered, the amplitude of the oscillations has gradually decreases until reaching the stationary state. The results confirm that the dissipation effect is large in the case of a large number of levels, see Fig. 1b. When combining qubit–qubit interaction ($R=5\lambda$) and neglecting dissipation ($\gamma =0$), in the case of a small number of levels, the lower values rise up and the amplitude of the oscillations decrease compared to the previous case. When the levels increase to 40, the period of collapse decreases, and the amplitudes of the vibrations decrease, as seen in Fig. 1c. When adding dissipation to the interaction, the function *W*(*t*) decreases slowly until it reaches a state of stability after a time longer than the previous case in (Fig. 1b). Moreover, the effect of dissipation resembles the previous case when the case of the number of levels is large, see Fig. 1d.

## Linear entropy

Entanglement has a pivotal role in quantum information (QI). However, it is a weak phenomenon in real experimental conditions because the quantum system is affected by the surrounding environment. These external influences lead to the disentanglement of parts of the system and eventually produce mixed quantum states^35^. The experimental and theoretical studies of quantum optical systems give a deep look at the links between mixedness and entanglement. The mixedness associated with a decrease in the quantum state purity is measured by linear entropy^36^, which is given by

18 $$\begin{aligned} P(t)= & 1-\left( \sum _{n=-j}^{j}|Y_{1}(n,t)|^{2}\right) ^{2}-\left( \sum _{n=-j}^{j}|Y_{2}(n,t)|^{2}\right) ^{2} \nonumber \\&-\left( \sum _{n=-j}^{j}|Y_{3}(n,t)|^{2}\right) ^{2}-\left( \sum _{n=-j}^{j}|Y_{4}(n,t)|^{2}\right) ^{2} \nonumber \\&-2\left| \sum _{n=-j}^{j}Y_{1}(n+1,t)Y_{2}^{*}(n,t)\right| ^{2}-2\left| \sum _{n=-j}^{j}Y_{1}(n+1,t)Y_{3}^{*}(n,t)\right| ^{2} \nonumber \\&-2\left| \sum _{n=-j}^{j}Y_{1}(n+2,t)Y_{4}^{*}(n,t)\right| ^{2}-2\left| \sum _{n=-j}^{j}Y_{2}(n,t)Y_{3}^{*}(n,t)\right| ^{2}\nonumber \\&-2\left| \sum _{n=-j}^{j}Y_{2}(n+1,t)X_{4}^{*}(n,t)\right| ^{2}-2\left| \sum _{n=-j}^{j}Y_{3}(n+1,t)Y_{4}^{*}(n,t)\right| ^{2} \end{aligned}$$

**Figure 2:**
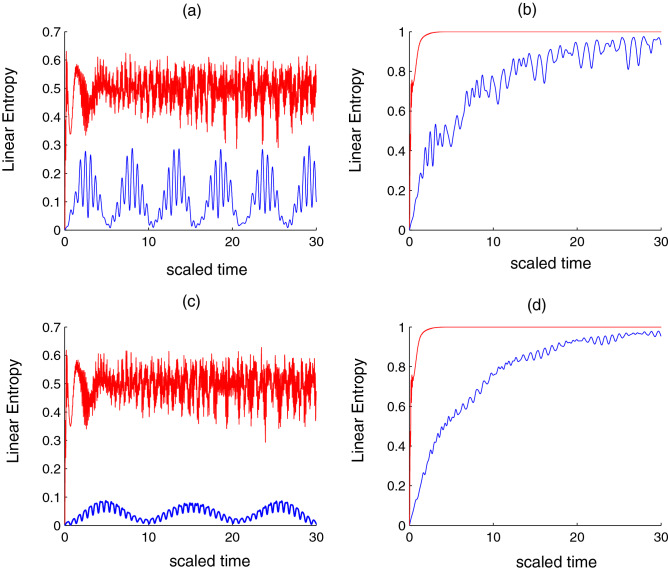
Time evolution of the *linear entropy* for the same conditions and parameters of Fig. 1.

Here we take the same conditions as the previous section to explore the linear entropy. When the number of levels is small, the dissipation and qubit–qubit interaction are neglected. Linear entropy has periodic oscillations and oscillates between 0 and 0.3. The *P*(*t*) function reflects weak entanglement and approaches zero regularly almost every (5.5)*n*, $n=0,1,2,...$ as seen from Fig. 2a. When the number of levels is large, the maximum and minimum values for linear entropy move upward. This reflects a strong entanglement between the su(2) system and the qubits. Moreover, the minimum values achieved at the beginning of the collapse period and the center of the revival period. The maximum values achieved at the beginning and end of the revival period, see Figs. 1a,2a. After takes the dissipation parameter into account, the *P*(*t*) function stabilizes to its maximum value. It is indicated that, when the number of levels is small, the *P*(*t*) function needs a long time to reach the stationary state, as shown in the Fig. 2b. When the qubit–qubit interaction is taken into account and dissipation is excluded, with regard to the small number of levels, the amplitude of previous fluctuations decreases and the linear entropy reaches the smallest values periodically. This phenomenon is repeated almost every 10*n*,  $n=0,1,2,..$. Whereas in the case of a large number of levels, the effect of the qubit–qubit interaction is very weak, see Fig. 2c. After combining the dissipation term, the oscillations fade quickly when the number of levels is large, while they fade slowly when the number of levels is small. The *P*(*t*) function needs more time to reach stability, as seen in Fig. 2d.

## Concurrence

The entanglement for bipartite systems has been represented by many measures. One of these measures is concurrence, which represents the approximate number of Bell pairs required to prepare a state using only local canonical transformations and classical communication. In two-qubit systems, the fully closed expression of concurrence is obtained by^37^

19 $$\begin{aligned} C_{Q}(t)=\max \{a_{1}-a_{2}-a_{3}-a_{4},0\},\;\;\; \end{aligned}$$

where $a_{k}$ $(k=1,2,3,4)$ are the eigenvalues of the square roots of the density matrix $R=\rho _{AB}(\sigma _{y}\otimes \sigma _{y})\rho _{AB}^{*}(\sigma _{y}\otimes \sigma _{y})$ and $\sigma _{y}$ is the Pauli matrix. $\rho _{AB}^{*}$ is the complex conjugate of $\rho _{AB}$. Concurrence vanishes, , namely $C_{Q}(t)=0$, for unentangled qubits, whereas $C_{Q}(t)=1$ , for maximally entangled qubits.

**Figure 3:**
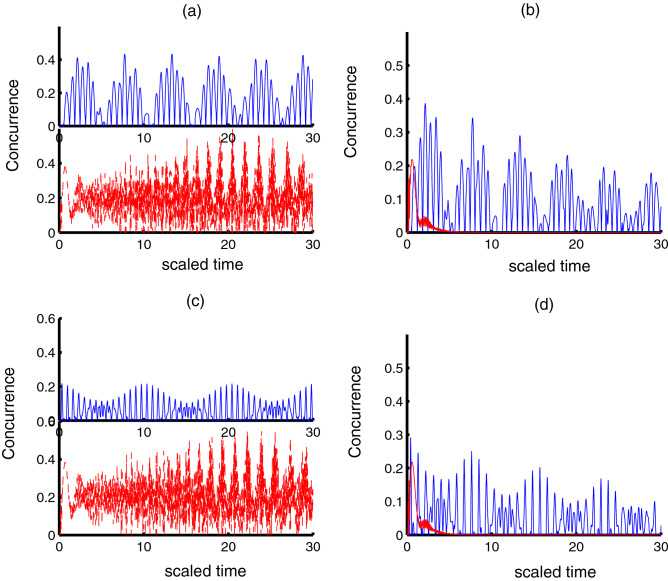
Time evolution of the *Concurrence* for the same conditions and parameters of Fig. 1.

With the same preconditions, we are now discussing the concurrence. We neglect the dissipation term and qubit–qubit interaction. When the number of levels is small, the concurrence fluctuates regularly between 0 and 0.4. In addition the phenomenon of sudden birth and sudden death are achieved at many points during the considered time. When the number of levels increases, the concurrence behavior changes completely, as the previous regular shape becomes random. The maximum value realized at the beginning of the collapse period and at the midpoint of the revival. The minimum values are realized at the beginning and end of the revival period, as shown in the Fig. 3a. It is indicated that, the values of the extreme values of the $C_{Q}(t)$ function are inversely proportional to the linear entropy by comparison of the behavior of the curves, as shown in Figs. 2a,3a. After taking the dissipation term into consideration, the oscillations of $C_{Q}(t)$ are quickly collapse and reach the minimum values in the case of a large number of levels, while the oscillations of the $C_{Q}(t)$ function decrease very slowly and need a long time until they reach the zero value. After the qubit–qubit interaction is included and dissipation term excluded, the amplitude of the vibrations decreases when the number of levels is small. The smallest values increase in the case of the number of levels is large and the phenomenon of sudden death and sudden birth almost disappears, as seen from Fig. 3c. After adding the dissipation, the $C_{Q}(t)$ function has the same behavior as before in the case of a large number of levels. While the amplitude of oscillations gradually decreases, but it needs a shorter time to reach the value of zero by comparing between Fig. 3b, d.

## Conclusion

An open system containing the interaction of the two qubits and a N-level atom is handled in this article. The multi-level atom is represented by su(2) algebra operator. In addition, the qubit–qubit interaction is considered. The constant of the motion is obtained, the matrix of time evolution operator is calculated; thus, we obtained the wave function depending on time explicitly. Through the wave function, the phenomena of collapse and revival are studied, the entanglement between the multi-level atom and the qubits is discussed using the linear entropy. In addition to discussing the entanglement of the two qubits together by the concurrence. Through the results in the case of a closed system ($\gamma =0$), it is found that the phenomenon of collapses and revivals did not appear when the number of levels is small, and the behavior of the population inversion is chaotic. In contrast, when the number of levels is large, one period of collapse appeared, followed by a period of revival, then there are random fluctuations. In the case of the open system ($\gamma =0.01\lambda$), the oscillations have more quickly decayed when having a large number of levels than when having a small number of levels. The qubit–qubit interaction resisted the decay of oscillations slightly and impeding the arrival of population inversion to a state of stability. The entanglement between the multi-level atom and the qubits increased in the case of a large number of levels and never reached the pure state. Adding the qubit–qubit interaction in the case of dissipation increased the time of the linear entropy and the concurrence to the state of stability. Moreover, a contrast in the behavior between the linear entropy and the concurrence without and with the dissipation term.

## References

[1] Yu, T. & Eberly, J. H. Quantum open system theory: bipartite aspects. *Phys. Rev. Lett.* **97**, (2006). 10.1103/PhysRevLett.97.140403 17155224

[2] Scully MO, Zubairy MS (2001). Quantum Optics.

[3] Agarwal GS (1974). Quantum Statistical Theories of Spontaneous Emission and their Relation to Other Approaches.

[4] Patsch, S., Maniscalco, S. & Koch, C. P. Simulation of open-quantum-system dynamics using the quantum Zeno effect. *Phys. Rev. Research* **2**, (2020).

[5] Lebreuilly, J., Chiocchetta, A. & Carusotto, I. Pseudothermalization in driven-dissipative non-Markovian open quantum systems. *Phys. Rev. A* **97**, (2018).

[6] Iotti RC, Rossi F (2020). Energy dissipation and decoherence in solid-state quantum devices: Markovian versus non-Markovian treatments. Entropy.

[7] Settineri, A., Macrí, V., Ridolfo, A. & Di Stefano, O. Dissipation and thermal noise in hybrid quantum systems in the ultrastrong-coupling regime. *Phys. Rev. A* **98**, (2018).

[8] Shahandeh, F., Lund, A. P. & Ralph, T. C. Quantum correlations and global coherence in distributed quantum computing. *Phys. Rev. A* **99**, (2019).

[9] Streltsov, A. *Quantum Correlations Beyond Entanglement and Their Role in Quantum Information Theory* (Springer, 2015).

[10] Adesso, G., Thomas, R. B. & Marco, C. Measures and applications of quantum correlations. *J. Phys. A* **49**, (2016).

[11] Berrada, K., Fanchini, F. F. & Abdel-Khalek, S. Quantum correlations between each qubit in a two-atom system and the environment in terms of interatomic distance. *Phys. Rev. A* **85**, (2012).

[12] Qureshi HS, Ullah S, Ghafoor F (2018). Hierarchy of quantum correlations using a linear beam splitter. Sci. Rep..

[13] Fu1, S., Luo, S. & Zhang, Y. Converting nonclassicality to quantum correlations via beamsplitters. *EPL (Europhys. Lett.)* **128**, 0295 (2020).

[14] Stefanov A, Zbinden H, Gisin N, Suarez A (2002). Quantum correlation with moving beamsplitters in relativistic configuration. Pramana.

[15] Liu, Y. X. *et al.* Qubit-induced phonon blockade as a signature of quantum behavior in nanomechanical resonators. *Phys. Rev. A* **82**, (2010).

[16] Hoffman, A. J. *et al.* Dispersive Photon Blockade in a Superconducting Circuit. *Phys. Rev. Lett.* **107**, (2011). 10.1103/PhysRevLett.107.053602 21867068

[17] Liu, Y., Xu, X., Miranowicz, A. & Nori, F. From blockade to transparency: controllable photon transmission through a circuit QED system. *Phys. Rev. A* **89**, (2014).

[18] Bose S, Jacobs K, Knight PL (1997). Preparation of Nonclassical States in Cavities with a Moving Mirror. Phys. Rev. A.

[19] Nielsen MA, Chuang IL (2000). Quantum Computation and Quantum Information.

[20] Raffah Bahaaudin (2020). Quantum correlations and quantum Fisher information of two qubits in the presence of the time-dependent coupling effect. Eur. Phys. J. Plus.

[21] Eberly JH, Yu T (2007). The end of an entanglement. Science.

[22] Abdel-Khalek S (2015). Quantum entanglement and geometric phase of two moving two-level atoms. Open Syst. Inf. Dyn..

[23] Abdel-Khalek S, Halawani SHA, Obada A-SF (2017). Effect of time dependent coupling on the dynamical properties of the nonlocal correlation between two three-level atoms. Int. J. Theor. Phys..

[24] Yu T, Eberly JH (2009). Sudden death of entanglement. Science.

[25] Bellomo B, Lo Franco R, Compagno G (2007). Non-Markovian effects on the dynamics of entanglement. Phys. Rev. Lett..

[26] Shore BW, Knight PL (1993). The Jaynes-Cummings Model. J. Mod. Opt..

[27] Dehghani, A., Mojaveri, B., Shirin, S. & Amiri Faseghandis, S. Parity deformed Jaynes-Cummings model: “Robust Maximally Entangled States”. *Sci. Rep.* **6**, 38069 (2016). 10.1038/srep38069 PMC5137026 27917882

[28] Miranowicz A, Leonski W (2004). Two-mode optical state truncation and generation of maximally entangled states in pumped nonlinear couplers. J. Opt. B.

[29] Kowalewska-Kudłaszyk, A. & Leoński, W. The phase of the coupling effect on entanglement decay in the nonlinear coupler system. *Phys. Scr.* (T140), 014050 (2010).

[30] Bartkowiak, M., Miranowicz, A., Wang, X., Liu, Y., Leoń ski, W. & Nori, F. Sudden vanishing and reappearance of nonclassical effects: General occurrence of finite-time decays and periodic vanishings of nonclassicality and entanglement witnesses. *Phys. Rev. A* **83**, 053814 (2011).

[31] Bose, I. & Chattopadhyay, E. Macroscopic entanglement jumps in model spin systems. *Phys. Rev. A* **66**, (2002).

[32] Sebawe Abdalla, M., Ahmed, M.M.A., Khalil, E.M. & Obada, A.-S.F. Dynamics of an adiabatically effective two-level atom interacting with a star-like system. *Prog. Theor. Exp. Phys.* **073A02** (2014).

[33] Chong, S. Y. & Shen, J. Q. Quantum collapse-revival effect in a supersymmetric Jaynes-Cummings model and its possible application in supersymmetric qubits. *Phys. Scr.* **95**, (2020).

[34] Khalil, E.M., Abdalla, M.S. & Obada, A.-S.F. Pair entanglement of two-level atoms in the presence of a nondegenerate parametric amplifier. *J. Phys. B, At. Mol. Opt. Phys.* **43**, 095507 (2010).

[35] Clark, S. G. & Parkins, A. S. Entanglement and entropy engineering of atomic two-qubit states. *Phys. Rev. Lett.* **90**, (2003). 10.1103/PhysRevLett.90.047905 12570464

[36] Jaeger, G. *et al.* Entanglement, mixedness, and spin-flip symmetry in multiple-qubit systems. *Phys. Rev. A* **68**, (2003).

[37] Wootters WK (1998). Entanglement of Formation of an Arbitrary State of Two Qubits. Phys. Rev. Lett..

